# Mechanisms and impact of the frequent exacerbator phenotype in chronic obstructive pulmonary disease

**DOI:** 10.1186/1741-7015-11-181

**Published:** 2013-08-14

**Authors:** Jadwiga A Wedzicha, Simon E Brill, James P Allinson, Gavin C Donaldson

**Affiliations:** 1 Centre for Respiratory Medicine, Royal Free Campus, University College London, Rowland Hill Street, Hampstead, London NW3 2PF, UK

**Keywords:** Chronic obstructive pulmonary disease (COPD), Exacerbations, Frequent exacerbator phenotype, Comorbidities

## Abstract

Exacerbations of chronic obstructive pulmonary disease (COPD) are important events that carry significant consequences for patients. Some patients experience frequent exacerbations, and are now recognized as a distinct clinical subgroup, the ‘frequent exacerbator’ phenotype. This is relatively stable over time, occurs across disease severity, and is associated with poorer health outcomes. These patients are therefore a priority for research and treatment. The pathophysiology underlying the frequent exacerbator phenotype is complex, with increased airway and systemic inflammation, dynamic lung hyperinflation, changes in lower airway bacterial colonization and a possible increased susceptibility to viral infection. Frequent exacerbators are also at increased risk from comorbid extrapulmonary diseases including cardiovascular disease, gastroesophageal reflux, depression, osteoporosis and cognitive impairment. Overall these patients have poorer health status, accelerated forced expiratory volume over 1 s (FEV1) decline, worsened quality of life, and increased hospital admissions and mortality, contributing to increased exacerbation susceptibility and perpetuation of the frequent exacerbator phenotype. This review article sets out the definition and importance of the frequent exacerbator phenotype, with a detailed examination of its pathophysiology, impact and interaction with other comorbidities.

## Introduction

Exacerbations of chronic obstructive pulmonary disease (COPD) are episodes of symptom worsening in COPD that have both short-term and longer-term consequences [1]. They are associated with increased airway and systemic inflammation and also increases in dynamic hyperinflation leading to classical exacerbation symptoms [2]. COPD exacerbations are major drivers of health status in COPD, and are important causes of hospital admission and death. Exacerbations are also key outcomes for therapies in COPD and thus there has been much progress in their understanding in recent times.

**Figure 1:**
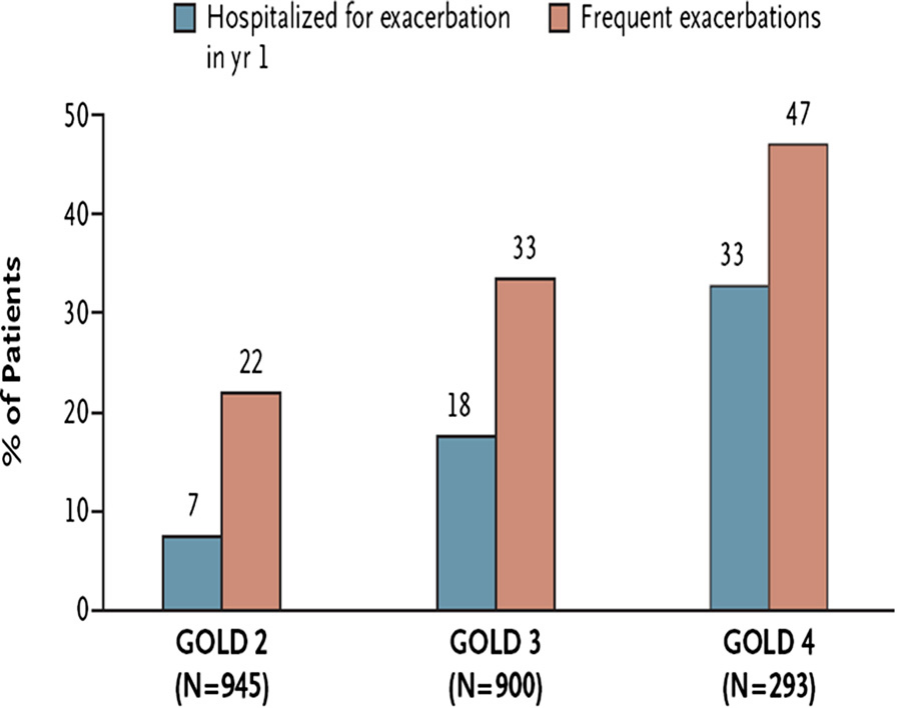
**Association of disease severity with the frequency and severity of exacerbations.** Reproduced from Hurst *et al*. [3] with permission.

**Figure 2:**
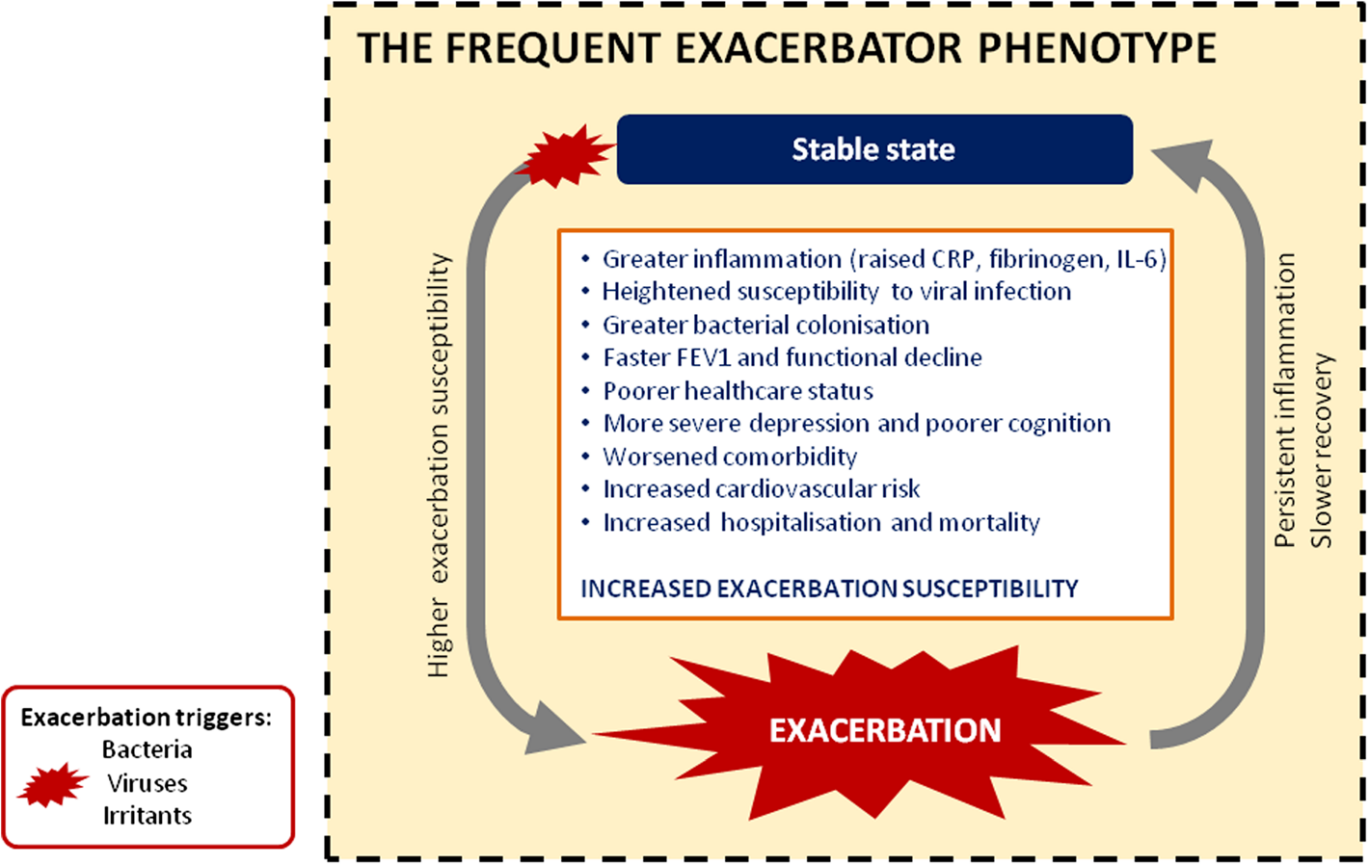
**Schematic illustration of the frequent exacerbator phenotype.** While many patients with chronic obstructive pulmonary disease (COPD) experience exacerbations, there is a subgroup of patients who enter a destructive cycle of frequent exacerbations with associated poorer outcome. This phenotype appears to be maintained over time.

It has also been recognized that some patients with COPD are particularly susceptible to exacerbations and these patients have been termed ‘frequent exacerbators’, in contrast to patients with infrequent exacerbations who experience few exacerbations over time. The frequent exacerbator phenotype has now been recognized as a major phenotype in patients with COPD and occurs across disease severities. The Evaluation of COPD Longitudinally to Identify Predictive Surrogate Endpoints (ECLIPSE) exacerbation study showed that even in patients defined as grade 2 according to the Global Initiative for Chronic Obstructive Pulmonary Disease (GOLD) (forced expiratory volume over 1 s (FEV1) 50% to 80% predicted), 22% of the COPD population were frequent exacerbators [3] (Figure 1). Therefore, this review will describe the pathophysiology and impact of the frequent exacerbator phenotype and will also address the interaction with comorbidity, as illustrated in Figure 2.

## Definition of COPD exacerbations and the frequent exacerbator phenotype

COPD exacerbations have been defined in two ways. Exacerbations are episodes of worsening of symptoms, and can be defined in terms of a persisting deterioration in respiratory symptoms outside normal day-to-day variation, In the London COPD cohort work [4], an exacerbation is defined as an increase of two major symptoms (either dyspnea, increased sputum purulence or increased sputum volume), or one major and one minor symptom (cough, wheezing, cold, nasal discharge, sore throat), sustained for 2 days or longer. Use of such a definition requires some form of daily monitoring with either a diary or an electronic device, but allows a complete count of all exacerbation events. It has been shown that exacerbations are often unreported and untreated and that these untreated exacerbations also affect health status [4-6]. Symptom measures for COPD exacerbations are therefore likely to be optimal for exacerbation detection in clinical trials [7].

Recently the EXACT-PRO (‘EXAcerbations of Chronic Pulmonary Disease Tool - Patient-Reported Outcome’) device has been developed as a validated outcome tool for COPD exacerbations and this is based on detecting changes in symptoms and thus registering events whether treated or untreated [8]. Epidemiological studies and clinical trials have both used healthcare utilization (HCU) as a measure of COPD exacerbations, though as explained above, these definitions are inaccurate, will vary with different healthcare systems, and will miss untreated events. Furthermore, many patients with COPD are slow to perceive exacerbation symptoms and thus will tend not to report exacerbations for therapy. It should also be noted that COPD exacerbations may mimic or coexist with other conditions, such as pneumonia, congestive cardiac failure, pneumothorax or pulmonary embolism, and thus it is important in clinical practice to accurately confirm the diagnosis and exclude other conditions.

Thus, the precise definition of the frequent exacerbator phenotype is problematic and will represent the number of exacerbations where a stable phenotype is present over time. The ECLIPSE exacerbation analysis showed that when a patient has two or more HCU exacerbations per year in 1 year, they have around a 68% chance of having two or more in the third year [3]. However, if both treated and untreated events are counted, as in the London cohort of patients with COPD, then the exacerbation rate for a stable phenotype will be larger. Further studies are required to address this issue.

## Pathophysiology of frequent COPD exacerbations

The heterogeneity of COPD exacerbations reflects their dependence on a complex spectrum of interacting factors. Susceptibility to exacerbations and the basis of the frequent exacerbator is therefore probably multifactorial. Identifying contributory factors, such as background airway inflammation, airway microbial patterns and host immunological responses, may provide potential targets in the effort to alter a patient’s exacerbation frequency phenotype.

### Airway and systemic inflammation at exacerbation and in the frequent exacerbator

During exacerbations of COPD the existing airway and systemic inflammation associated with this inflammatory disease increases further [9-14]. An inflammatory cascade releases inflammatory mediators, such as interleukins and chemokines, which recruit and activate immune cells. This cascade is thought to contribute to the local structural damage responsible for COPD progression, but also causes systemic inflammation with increases in acute phase proteins such as fibrinogen [11] and C reactive protein (CRP) [15]. Although the exacerbation inflammatory process is predominantly neutrophilic [16,17], the numbers of all inflammatory cell types within bronchial mucosa increase and the inflammatory character is also influenced by the type of insult triggering an exacerbation. For example, compared to bacterially driven exacerbations viruses appear to stimulate more eosinophil activity [17,18].

The airways of frequent exacerbators, however, appear to be more inflamed with higher levels of sputum interleukin (IL)-6 and IL-8 even in the stable state [9]. In addition, their trajectory of inflammation is also worse with sputum IL-6 and plasma fibrinogen [19] continuing to increase more rapidly over time.

Frequent exacerbators have higher sputum IL-6 and serum CRP during their exacerbation recovery periods [20], making persistent post-exacerbation inflammation a possible explanation for their higher baseline inflammation. A higher CRP during the recovery period is also associated with a shorter time until the next exacerbation [20] perhaps suggesting an inflammatory basis to the temporal clustering of exacerbations reported by Hurst *et al*., who identified the 8-week post-exacerbation period as particularly high risk for exacerbation recurrence [21].

Persisting and increased propensity to inflammation therefore is a feature of the frequent exacerbator phenotype, although whether these features are causative or are downstream of susceptibility to the triggers of exacerbations remains to be established.

### Mechanical factors

Small airway obstruction in COPD is accompanied by dynamic lung hyperinflation and this is a major driver of respiratory symptoms, particularly dyspnea [22]. At exacerbation, airway inflammation and worsened expiratory flow limitation worsen this, leading to further dynamic hyperinflation with increased respiratory effort, cardiovascular strain, inspiratory muscle overload and potential respiratory failure [2]. In those patients with severe airflow obstruction and a lower FEV1% predicted, hyperinflation is more likely to be present even in stable disease and there may be reduced capacity to deal with any further insult. Exacerbations would potentially be more easily triggered in these patients and this may explain why a higher exacerbation frequency is seen with more severe airflow restriction.

Bronchodilator therapy, by improving small airway obstruction, has been shown to reduce lung hyperinflation in stable disease and therefore may partially ‘reset’ the threshold at which exacerbation occurs [23]. This provides a mechanism by which bronchodilator therapy may reduce exacerbation frequency even in the absence of any anti-inflammatory effects.

### Triggers of COPD exacerbations

Most exacerbations appear to be associated with infective triggers, either bacterial or viral, although ‘non-infective’ triggers such as air pollution may also be important. Most of the 40% of acute exacerbations linked to viral infections involve rhinovirus (58%) although other respiratory viruses implicated include human respiratory syncytial virus, coronavirus, influenza virus, parainfluenza virus and adenoviruses [24,25]. These viral associated exacerbations exhibit greater systemic or airway inflammation, greater symptom burden and longer recovery times [9,11,24].

### Susceptibility to viral infection, COPD and the frequent exacerbator phenotype

An increased viral susceptibility has been proposed as a cause of more frequent exacerbations in COPD [25]. Cigarette smoking does appear to increase viral susceptibility [26,27] but whether or not the overall COPD population itself is more susceptible is disputed. Data from an analysis in the London COPD cohort showed that upper airway colds are more likely to occur in patients with a history of frequent exacerbations, suggesting that these patients have enhanced susceptibility to viral infection [28]. There is also evidence that patients in whom a respiratory virus is detected have a higher exacerbation frequency [24]. Monto *et al*., in an early study, demonstrated that patients with chronic bronchitis had a greater incidence of serologically proven rhinovirus infection [29] although this work involved small study groups, lacked spirometric measurements and preceded molecular viral detection techniques. Further support for the viral susceptibility theory came from Wald *et al*., who studied a rhinovirus outbreak within a care facility and found that patients with COPD were over-represented in the infected group [30]. However, contradicting these findings is the work by Greenberg *et al*. who, again using small groups, found no excess incidence of viral respiratory infections in patients with COPD versus controls, though frequent exacerbators were not specifically studied in this paper [31]. Well-powered studies are required to firmly establish whether the frequent exacerbator phenotype in particular experiences a greater proportion of viral exacerbations than infrequent exacerbators.

There are a number of proposed mechanisms of increased viral susceptibility including modulation of intracellular adhesion molecule 1 (ICAM-1). Most rhinovirus serotypes (major group) attach to respiratory epithelial cells by binding to ICAM-1 [32] and through this transmembrane protein modulate the recruitment and activation of inflammatory cells. Increased ICAM-1 increases susceptibility to infection and ICAM-1 appears to be upregulated in the bronchial mucosa of patients with chronic bronchitis [33].

While viruses are separately thought to be a major cause of COPD exacerbations, 25% of exacerbations involve coinfection with viruses and bacteria. These exacerbations exhibit greater functional impairment, with longer hospitalization [17,24], and are associated with higher airway bacterial load and greater airway inflammation [34]. Recent work using rhinovirus inoculation as an experimental model of COPD exacerbation shows a consistent increase in bacterial numbers following the initial viral infection [35] and this supports the hypothesis that bacterial exacerbation may be precipitated by viruses.

### Bacterial infection and COPD exacerbations

Common bacteria associated with many COPD exacerbations include *Streptococcus pneumoniae*, *Haemophilus influenzae*, *and Moraxella catarrhalis*[36] and their presence at exacerbation is accompanied by greater levels of systemic inflammation [37]. Besides the introduction of these bacterial species or an increase in their bacterial load, it has also become clear that even a change in bacterial strain can trigger exacerbations [38].

In the absence of active infection, the identification, by traditional culture methods, of bacteria in the lower airway has been termed ‘colonization’. This term gives the illusion that such bacterial presence is benign, yet it is actually associated with greater inflammation, poorer lung function [37], chronic bronchitis [39-41], and more frequent exacerbations [42].

With the development of new molecular techniques it is clear that even the healthy lower airway is not sterile [43] and so we must re-evaluate the meaning of previously identified colonization and the mechanism by which it influences exacerbation frequency. Colonization may actually represent a disturbance of the normal microbiome, with subsequent overgrowth of a particular species, which drives the inflammatory process in COPD. Alternatively, it may be the inflammatory process that impairs normal regulation of bacterial presence making colonization a marker and potential amplifier of underlying inflammatory damage rather than its primary driver. Longitudinal studies are required to establish how the airway microbiome changes at exacerbation and how this relates to colonization and the frequent exacerbator phenotype.

### Genetic predisposition of exacerbation susceptibility

The existence of a seemingly distinct exacerbator phenotype raises the question of whether there is a genetic predisposition towards frequent exacerbations in COPD. This question has not yet been clearly answered, although some distinct gene polymorphisms appear to be linked to exacerbation frequency. Takabatake and colleagues [44] reported that a single nucleotide polymorphism in the CCL-1 gene, encoding a leucocyte chemotactic factor, was predictive of the frequency and severity of exacerbations, while polymorphic variation in surfactant protein B has also been linked with exacerbation frequency [45]. Mannose-binding lectin (MBL) deficiency is associated with susceptibility to respiratory infections; a higher frequency of MBL gene polymorphisms was noted in patients with COPD who experience frequent exacerbations [46] and may also predict hospital admission for COPD [47]. In addition to these, an increased exacerbation frequency was found in patients whose sputum contained markers suggestive of increased microsatellite DNA instability, suggesting that those patients with a higher DNA mutation rate experience more frequent exacerbations [48]. Further research is needed to explore this further.

### Adherence to medications

It has been suggested that half of all patients do not take their medications adequately [49], and these patients may be missing the benefit of therapies that would otherwise reduce exacerbation frequency. Treatments for COPD are varied, including rehabilitation, smoking cessation, oxygen therapy as well as medications administered via different routes and devices, and adherence is therefore complex and difficult to measure; it may differ between therapies and even in individual patients [50]. However, patients who are non-adherent to COPD therapy experience more frequent hospitalizations [51] and have greater healthcare utilization than those who adhere [52], and therefore non-adherence may be another etiological factor in the development of the frequent exacerbator phenotype. Factors linked to non-adherence include age, complexity of treatment regimen, nature of inhaled delivery device, patient education, and importantly depression [53], which is itself common in COPD and linked to exacerbation frequency as described below.

## Impact of frequent exacerbations in patients with COPD

### Lung function decline

Over the last 15 years, the importance of frequent exacerbations on disease progression and patient reported outcomes has become increasingly apparent. Exacerbations were once considered to be of little or no importance. This was based on the pivotal 10-year study by Fletcher and colleagues, which found no relationship between lung function decline and chest infections [54]. However, their patient cohort had only limited airflow obstruction. In patients with moderate to severe COPD, two studies initially showed that frequent exacerbations accelerate lung function decline. Donaldson and colleagues [55] reported a 25% faster decline in FEV1 (-32.1 mL/year vs -40.1 mL/year; *P* <0.05) in patients with frequent exacerbations. Kanner and colleagues [56] reported on patients with mild COPD who continued to smoke; in this group, experiencing 1.5 lower respiratory tract illnesses per year was associated with a decline in FEV1 of -69.4 ml/year, compared to a decline of -55.9 ml/year. Larger interventional or observational studies have subsequently confirmed these findings and the effect of exacerbations on disease progression [57-61].

### Quality of life

It has been known for some time that frequent exacerbations have a profound impact on the health status of patients with COPD. Seemungal and colleagues reported that patients with three or more exacerbations per year (both treated and untreated) had a poorer health-related quality of life compared patients with zero to two exacerbations per year, as measured with the disease specific St. Georges Respiratory Questionnaire (SGRQ) [4]. Recently, other measures of health status, such as the COPD assessment test (CAT), have shown a worse score in frequent exacerbators with >2 exacerbations per year of 19.5 compared to 16.8 in patients with <2 exacerbations per year [62].

The impact of exacerbations as assessed by the SGRQ in a study also persists for some time after the event, as patients only approached baseline within the next 6 months [63]. Other assessment measures show faster rates of improvement, and in patients who did not relapse with another exacerbation, significant improvements were seen in the four domains of the Chronic Respiratory Questionnaire (CRQ) between presentation and 10 days later [64].

Feelings of fatigue are also impacted with the Functional Assessment of Chronic Illness Therapy-fatigue (FACIT-fatigue) scale, showing worsening of fatigue at exacerbation and worsening of scores with increasing exacerbation frequency [65].

### Activity

Peripheral muscle weakness is associated with COPD exacerbations, and muscle force (quadriceps peak torque) was found to be lower at 3 days after admission for an exacerbation than 90 days later [66]. An acute fall in outdoor activity is also seen at exacerbation [67] and a number of factors may contribute: physical inactivity, bed rest, metabolic, nutritional and inflammatory status and steroid treatment [68]. Exacerbations also appear to have a permanent effect on physical activity; patients with frequent exacerbations (>2.47 per year) have a faster decline in time outdoors each day of -0.17 h/year (*P* <0.011) in addition to the decline of -0.10 h/year that occurs in infrequent exacerbators [67].

### Hospital admissions

Patients with a history of frequent exacerbations are those also most likely to be admitted to hospital. In the ECLIPSE exacerbation study, patients with GOLD stage 2 COPD (FEV1 50% to 80% predicted) already had a 7% admission rate to hospital for COPD exacerbation-related causes over the first year of follow-up, and this increased to 33% of patients admitted over a year with stage 4 disease (FEV1 less than 30% predicted) [3]. There is evidence that delayed therapy for an exacerbation leads to longer exacerbations and a higher chance of hospital admission. Thus, patients must be instructed about symptom recognition and the importance of early intervention [5].

### Mortality

There is a wide range reported of inpatient mortality from COPD exacerbations, which reflects comorbidity and admission practices. Risk factors for inpatient mortality include cor pulmonale, congestive heart failure, a low arterial pH, leg edema, age, oxygen saturations <86%, assisted ventilation and low body mass index [69,70]. A UK audit showed that larger general hospitals and teaching hospitals also have better mortality rates than small district general hospitals [71].

Frequent acute exacerbations are also an independent risk factor for all-cause mortality in COPD [72], and every hospitalized exacerbation worsens disease progression. The risk of death is increased by up to five times following the tenth admission compared to the first [73].

## Exacerbation frequency and comorbidities

### Cardiovascular comorbidity

Cardiovascular diseases form the largest group of comorbidities seen in patients with COPD, with ischemic heart disease (IHD) the most important condition. Prevalence estimates vary; a pooled analysis of 2 large epidemiological studies involving 20,296 patients in the USA [74] reported a prevalence of 20% to 22% in patients with COPD compared to 9% in controls, with even symptomatic patients with early COPD with normal spirometry at increased risk. Patients with comorbid IHD in the London COPD cohort had longer exacerbations, worse health status, more dyspnea and a lower exercise capacity than those without [75].

Systemic inflammation, a predominant feature of COPD, is strongly associated with cardiovascular risk. Chronic inflammation is associated with the development and rupture of atherosclerotic plaques [76], and C reactive protein, elevated in patients with COPD, predicts cardiovascular events [77]. Levels of the inflammatory marker IL-6 are also higher in patients with COPD with cardiovascular disease [78] and frequent exacerbators have higher levels of IL-6 and fibrinogen than infrequent exacerbators [19]. Systemic inflammation contributes to a prothrombotic state, increasing the likelihood of cardiovascular and thromboembolic events [79]. Cardiovascular risk is also linked to vascular dysfunction. Large vessel arterial stiffness strongly predicts cardiovascular events and mortality [80] and is raised in patients with COPD [81], while small vessel dysfunction is suggested by higher levels of microalbuminuria [82] and cerebral small vessel disease [83]. Hypertension, a key risk factor for further cardiovascular events, is probably also linked to vascular dysfunction and is described in 40% to 60% of patients with COPD compared to 34% of controls [74].

**Figure 3:**
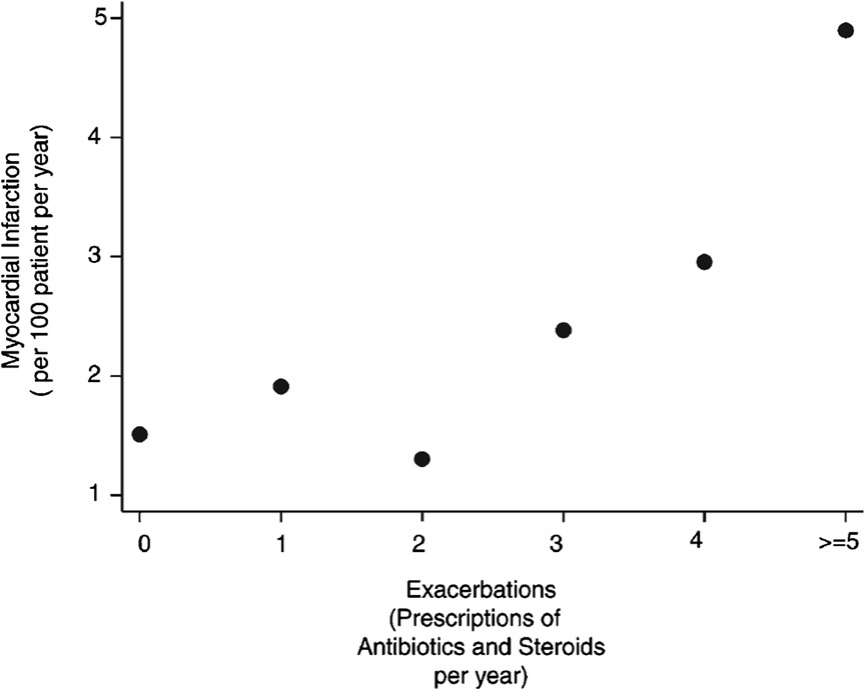
**Annual rate of myocardial infarction against the annual rate of exacerbation (defined as prescription of steroids and antibiotics together).** Reproduced from Donaldson *et al*. [86] with permission.

Exacerbations have a profound and direct impact on patients’ cardiovascular status. Of 242 patients hospitalized for COPD exacerbation in Scotland, 12% were found to meet universally agreed criteria for the diagnosis of myocardial infarction with raised troponin and either chest pain or serial electrocardiogram (ECG) changes [84]. The period following an exacerbation appears to be particularly high risk. In the 4-year UPLIFT trial, the relative risks of cardiac failure, myocardial infarction and stroke were 10.71, 3.20 and 2.31 respectively, in 2,289 patients, for the 180 days after their first exacerbation compared to the 180 days preceding it [85]. In addition, evidence from a large observational database in the UK showed a 2.27-fold increased risk of clinical myocardial infarction in the first 5 days after an exacerbation and a 1.26-fold increased risk of stroke in the first 49 days afterwards [86]; patients who experienced myocardial infarction or stroke had a higher yearly exacerbation frequency than those who did not (Figure 3). Pulmonary embolism may be also associated with COPD exacerbations, though the reported prevalence of pulmonary embolism is variable [87]. However, patients with exacerbations complicated by pulmonary embolism have a poorer outcome [79].

Treatment of cardiovascular disease and hypertension is therefore a priority for patients with COPD, especially frequent exacerbators. Secondary prevention for cardiovascular events usually involves antiplatelet agents, statins, β-blockers and angiotensin-converting enzyme inhibitors, and there is evidence that these reduce mortality in COPD [88-90]. Cardioselective β-blockers appear safe and beneficial in COPD [91,92] and may also reduce admissions due to exacerbations [93]. Low-molecular-weight heparin is effective at reducing the risk of venous thromboembolism in patients hospitalized with respiratory illness [94] and should be considered in all inpatients with COPD exacerbation.

### Gastroesophageal reflux disease (GORD)

GORD is present in up to 30% to 60% of patients with COPD [95] and there is growing evidence to suggest that it plays an important role in exacerbations. Two studies have reported a higher exacerbation frequency in patients with GORD symptoms [96,97], and the large ECLIPSE study showed an association between self-reported GORD and having ≥2 treated exacerbations per year [3]. Although the etiology remains unclear, there is a plausible causative mechanism by way of microaspiration of stomach contents causing increased airway inflammation and potentiated by the effect of hyperinflation common in COPD. The reflux is complex, with non-acid and gaseous reflux likely to be prominent in COPD. This potentially represents a novel therapeutic target, and a single-blind trial of 100 patients with COPD showed a significant reduction in exacerbations from 1.18 to 0.32 over 1 year of treatment with lansoprazole 15 mg daily [98] despite excluding patients with clinically overt GORD. Further research is needed into the causes and mechanisms of GORD in COPD and whether other agents modifying gastric motility may be more appropriate interventions.

### Neuropsychiatric complications

Depression is common in patients with COPD; the ECLIPSE study found a prevalence of symptom-defined depression in 26% of patients compared to 12% of controls, increasing with disease severity [99]. There is a clear relationship to exacerbation frequency, and frequent exacerbators have higher depression scores even at baseline; patients who are depressed have worse breathlessness and quality of life, are more socially isolated and spend less time outdoors [100]. Depression in COPD is also linked to poorer outcomes including hospital readmission and all-cause mortality [101,102]. Given that depression is a risk factor for poor engagement with many aspects of healthcare, including medication adherence [103] and completion of pulmonary rehabilitation programs [104], these patients may receive inadequate treatment for their COPD and this may further contribute to the poor outcomes above. Depressive symptoms should be recognized and treated alongside the underlying COPD.

Impaired cognitive function is increasingly recognized in COPD, at exacerbation in particular. A recent prospective study showed that patients with COPD had significantly worse cognitive function than controls, with those who had just recovered from an exacerbation significantly worse than those at stable state [105]. Crucially, there was no recovery at 3 months. This suggests that exacerbations are associated with sustained cognitive dysfunction, and frequent exacerbators may therefore be subject to more rapid cognitive decline.

### Osteoporosis

Osteoporosis is common, with a reported prevalence of 35% in patients with COPD [106], and is related to the degree of emphysema seen on computerized tomography (CT) scan [107]. These patients are often already at increased risk due to comorbidities and relative inactivity, and this prevalence is not fully explained by corticosteroid use alone [106]. Thoracic vertebral compression fractures due to osteoporosis cause back pain and worsening kyphosis and are associated with a decrease in vital capacity (previously quantified as 9% per fracture [108]). Frequent exacerbators have a much larger annual decrease in bone mineral density compared to infrequent exacerbators (5.41% vs 0.60%) [109], and are therefore more likely to be susceptible to fractures. Therefore, these patients should be carefully investigated for osteoporosis and treated as appropriate.

## Conclusions

This review has described the impact of the frequent exacerbator phenotype on patients with COPD and their considerable morbidity. Frequent exacerbators can be identified across the COPD disease spectrum and must be targeted for effective exacerbation prevention, both with pharmacological and non-pharmacological agents. Susceptibility to respiratory viral infection is likely to be an important mechanism in frequent exacerbators and novel interventions need to be developed to reduce viral infection at exacerbation. There is now some evidence that interventional therapy with an anti-inflammatory agent can modify the frequent exacerbator phenotype such that patients become infrequent exacerbators [110].

By understanding the mechanisms of development of frequent exacerbations, and applying appropriate interventions, we will be at last able to impact on the health status of this high-risk patient group.

## Abbreviations

CAT: COPD assessment test; COPD: Chronic obstructive pulmonary disease; CRP: C reactive protein; CRQ: Chronic respiratory questionnaire; CT: Computerized tomography; ECLIPSE: Evaluation of COPD longitudinally to identify predictive surrogate endpoints; FACIT-Fatigue: Functional assessment of chronic illness therapy-fatigue scale; FEV1: Forced expiratory volume over 1 s; GOLD: Global initiative for chronic obstructive pulmonary disease; GORD: Gastroesophageal reflux disease; ICAM: Intracellular adhesion molecule; IHD: Ischemic heart disease; IL: Interleukin; MBL: Mannose-binding lectin; SGRQ: St George’s respiratory questionnaire.

## Competing interests

JAW has received honoraria for lectures and/or advisory boards from GSK, Boehringer, Bayer, Novartis, Vifor Pharma, Chiesi, Pfizer, Takeda and Almirall. JAW has also received research grant funding from Takeda, GSK, Novartis and Chiesi. SEB, JPA and GCD have no competing interests to declare.

## Authors’ contributions

All the authors contributed to the writing of the review and performed the literature searches. All authors were involved at all stages of the editing process and have seen and approved the final manuscript.

## Pre-publication history

The pre-publication history for this paper can be accessed here:

http://www.biomedcentral.com/1741-7015/11/181/prepub
